# Non-Destructive Identification of Fibre Orientation in Multi-Ply Biaxial Laminates Using Contact Temperature Sensors

**DOI:** 10.3390/s20143865

**Published:** 2020-07-10

**Authors:** David I. Gillespie, Andrew W. Hamilton, Ewan J. McKay, Brian Neilson, Robert C. Atkinson, Ivan Andonovic, Christos Tachtatzis

**Affiliations:** 1 Department of Electronic and Electrical Engineering, University of Strathclyde, Royal College Building, 204 George Street, Glasgow G1 1XW, UK; andrew.w.hamilton@strath.ac.uk (A.W.H.); robert.atkinson@strath.ac.uk (R.C.A.); i.andonovic@strath.ac.uk (I.A.); christos.tachtatzis@strath.ac.uk (C.T.); 2 Collins Aerospace, Prestwick, 1 Dow Avenue, Prestwick International Aerospace Park, Ayrshire KA9 2SA, UK; ewan.mckay@collins.com (E.J.M.); Brian.neilson@collins.com (B.N.)

**Keywords:** aerospace, composite, inspection, maintenance repair overhaul, non destructive inspection, quality assurance, re-manufacture, thermography

## Abstract

Fibre orientation within composite structures dictates the material properties of the laminate once cured. The ability to accurately and automatically assess fibre orientation of composite parts is a significant enabler in the goal to optimise the established processes within aftermarket aerospace industries. Incorrect ply lay-up results in a structure with undesirable material properties and as such, has the potential to fail under safe working loads. Since it is necessary to assure structural integrity during re-manufacture and repair assessment, the paper demonstrates a novel method of readily and non-destructively determining fibre orientation throughout multi-ply Biaxial woven composite laminates using point temperature contact sensors and data analysis techniques. Once cured, only the outermost laminates are visible to assess orientation. The inspection method is conducted visually, with reference guides to allow for rapid adoption with minimum training, as well as harnessing established temperature sensors within the Maintenance Repair and Overhaul (MRO) environment. The system is amenable to integration within existing repair/re-manufacture processes without significant impact to process flow. The method is able to identify noisy samples with an accuracy, precision and recall of 0.9, and for synthetically created samples of double the cure ply thickness, a precision of 0.75, a recall of 0.7 and an accuracy of 0.87.

## 1. Introduction

Low cost temperature sensors and low temperature heat sources form the basis for the implementation of a method to identify differences in thermal profiles of composites from low resolution data capture [1]. Within the Maintenance Repair and Overhaul (MRO) aerospace operational environment, the demand is not only to provide quality assurance of executed repairs, but to identify and characterise repairs executed by third parties for which there are often partial or no supporting records. The repair of composite structures requires removal of the damage and surrounding areas via cutting and drilling. The optimal performance of machine tooling used in processing Carbon Fibre Reinforced Plastic (CFRP) is application specific governed by the fibre characteristics within the structure [2].

The fibre orientation within a composite part has an impact upon the failure rate of Fibre Reinforced Plastic (FRP) laminate structures undergoing cyclic loading [3]. Non-symmetrical ply lay-ups create cured laminates with deformations that vary with fibre orientation symmetry [4,5,6]. In thin laminates, deformations are visible due to the magnitude of strain within the structure. Stiffness of the laminate—the resistance to bending—is increased by thickening the structure [7] or through the creation of a sandwich of two laminates bonded to either side of a stiffener, such as a honeycomb core [8]. Consequently visual inspection for bending due to the symmetry of laminate plies on thick and sandwich composite structures are unlikely to indicate non-symmetrical lay-ups.

X-ray computed tomography is an industry standard technique—amenable to enhancements in terms of information content through machine learning algorithms—that identifies individual fibre orientations within laminate structures and provides an indication as to the mean orientation of the laminate [9,10,11]. Results can be obtained quickly with a high degree of accuracy and level of detail for samples within the scan area. However for larger structures, several images are required to be stitched together to produce a full scan. Owing to the safety consideration associated with the use of X-rays in respect to human operators, the inspection equipment is ideally automated with either (i) static samples and moving scanners, (ii) static scanners and moving samples or (iii) a hybrid, the use of X-ray computational tomography becomes a time consuming and expensive process. The MRO environment differs to that of an Original Equipment Manufacturer (OEM), especially in respect to the nature of the structure condition. An alternative inspection method is Infra Red (IR) Thermography, it is well established that IR Thermography is able to provide accurate and reliable indications of fibre orientation [12,13,14,15,16,17,18]; however, Infrared Thermogrphic techniques rely on a consistent surface emissivity [19]. OEM parts are generally constructed from new materials and as such are clean and free from contaminants. MRO structures originate from in-service aircraft and are often subject to differing degrees of repair, including modifications to the surface e.g., paint removal and surface treatments. As such this technique produces variable results under these conditions.

A modelling approach to characterising the thermal profile of Biaxial laminates and identify ply orientation, is to rely entirely on finite element analysis. However, reported research has demonstrated that the transient thermal interactions are more complex at the ply boundaries and when simplified the resultant models are limited [1].In particular, transient thermal simulations through finite element analysis concluded that two ply Biaxial laminate samples produce a balanced thermal spread in the *x*- and *y*-axis along the length of the fibres for 0/90/90/0 and 0/90/0/90 samples [1]. Changes of 90 degrees to the fibre orientation at this boundary result in a significant change to the thermal profile on the far side of a sample in terms of thermal transmission. Contact temperature sensors for cure monitoring such as thermocouples and Resistance Temperature Detectors (RTDs) are in routine use in composite repair processes and as such the devices, configuration and measurements are well understood and accepted by operators. The proposed approach will utilise existing cure monitoring sensors and heat sources in order to generate thermal profile data, adopting the principles of traditional IR thermography but implemented through contact temperature sensors.

As the existing range of techniques, which can accurately and reliably produce indications of fibre orientation, currently require equipment and training that is not immediately available within most MRO facilities, there is a financial motivation for the need of a new method to inspect composite structures utilising existing equipment with minimal training. The method makes use of low cost contact temperature sensors and low temperature heater mats, currently used in the composite repair process within MROs, to identify fibre orientation within the repair regions of composite parts. This enables aerospace MRO providers to deliver services in a safer, faster and scalable manner.

## 2. Materials and Methods

**Figure 1:**
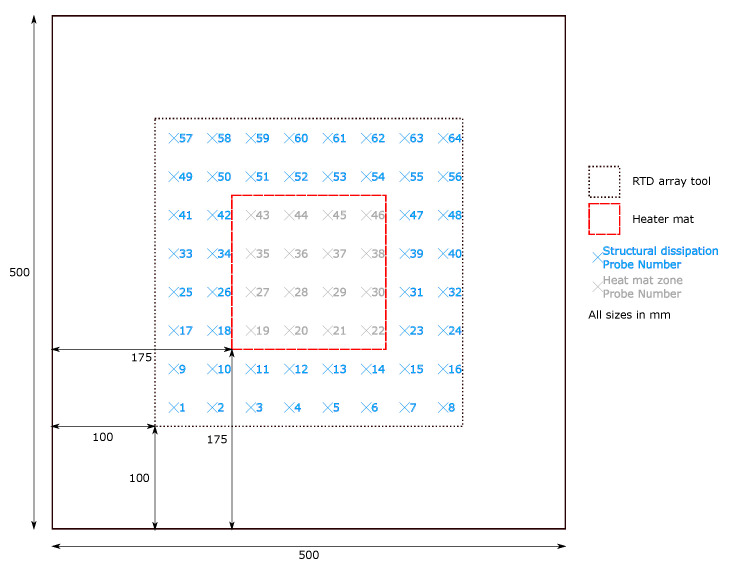
The 500 by 500 mm sample panel data capture set up with 150 mm × 150 mm heat zone (dashed red) and 300 by 300 mm RTD probe array (dashed black).

In total, 3 Biaxial woven aerospace-grade five harness CFRP pre-impregnated laminates were produced in 500×500 mm samples to prove the feasibility of the method. Each laminate comprised two ply samples of 1589 kg m${}^{-1}$ density and the thermal properties described in Table 1. These were laid up with three differing fibre orientations; 0/90/90/0 (symmetrical and balanced); 0/90/0/90 (non-symmetrical and balanced); and 0/90/+45/−45 (non-symmetrical and unbalanced). The tool to obtain thermal conduction profiles across the samples comprised two elements; 64 Resistance Temperature Detector (RTD) array with sensors spaced at 37.5 mm intervals within a 300×300 mm grid; and a 150×150 mm Proportional Integral Derivative (PID) controlled heat mat (Figure 1). The heat mat was applied to the bottom surface of the sample under inspection and the RTD probe array applied to the top surface. The samples were orientated such that the top surface in contact with the RTD array displayed the 0/90 fibre orientation, with the rear surface of the sample in contact with the heat mat varying between 0/90, 90/0 and +45/−45 depending on the sample.

The thermal stimulus following a step profile within the heat zone (Figure 1) is outlined in Table 2. The stimulus period was 5 minutes to allow sufficient time for the transient thermal profile to develop and the thermal energy to conduct and dissipate.

The temperature recorded on the top surface of the laminate can be attributed to two key thermal transfer processes. The area directly above the heat zone is the result of thermal transmission though the laminate, where as areas outside the heated zone experience temperature changes due to the compounding effect of transmission and thermal conduction in the direction of the fibres. The temperature change (ΔT) was recorded over time (*t*) for all temperature probes. The probes positioned directly above the heat zone do not show significant variations between samples that permit the discrimination of ply lay-ups. However, probes positioned in the the areas outwith the heat zone are primarily impacted by thermal conduction through the fibres and consequently permit the differentiation of fibre orientation and ply lay-up.

## 3. Analysis of Results

**Figure 2:**
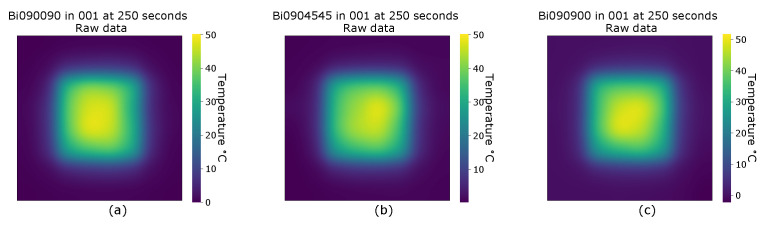
Heat maps of samples at 80 ${}^{\circ }$C and 250 seconds, (**a**) Biaxial 0/90/0/90, non symmetrical and balanced, (**b**) Biaxial 0/90/+45/−45, non symmetrical and unbalanced, (**c**) Biaxial 0/90/90/0, symmetrical and balanced.

Initial analysis of the samples was conducted through heat maps to identify significant visual differences in the thermal profile. Variation over the heated area is evident on comparison of all three samples at corresponding time intervals (see Figure 2). However such variations that may be observed produce patterns which are subjective and as such, are not sufficiently robust as an inspection method alone. Furthermore, no readily apparent differences are visible between maps outwith the heat zone and temperature changes within the dissipation area are attributed to the thermal conduction along the fibres. However, from observation, the thermal spread is governed by the fibre directions, manifest by the temperature changes (ΔT) outwith the heated area varying between 0 ${}^{\circ }$C and approximately 12 ${}^{\circ }$C. Consequently further analysis will concentrate on the regions where the temperature differences fall within that range highlighted in blue on Figure 1.

A Kernel Density Estimate (KDE) was used to generate the probability density function as a function of temperature change in ${}^{\circ }$C and to present the data in a more visually descriptive manner, easing the initial inspection. KDE was selected due to the ability to provide readily interpreted visualisation of quantitative data [20]. A bandwidth of 1, selected through trial and error, was used to achieve a constant bandwidth between samples that prevented over-smoothing of the data and allowed the accentuation of features.

**Figure 3:**
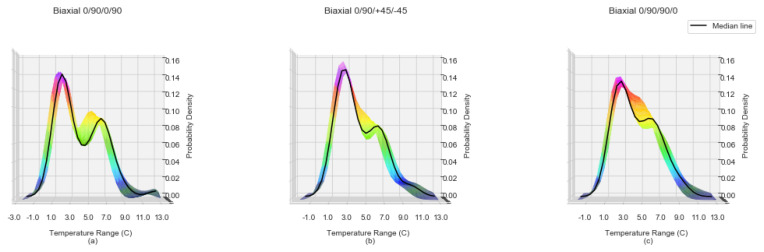
Kernel density estimates with median line for Biaxial laminate samples for temperature changes ranges −3${}^{\circ }$ to 12${}^{\circ }$ for the time period 150 to 300 s (**a**) Biaxial 0/90/0/90, non symmetrical and balanced, (**b**) Biaxial 0/90/+45/−45, non symmetrical and unbalanced, (**c**) Biaxial 0/90/90/0, symmetrical and balanced.

**Figure 4:**
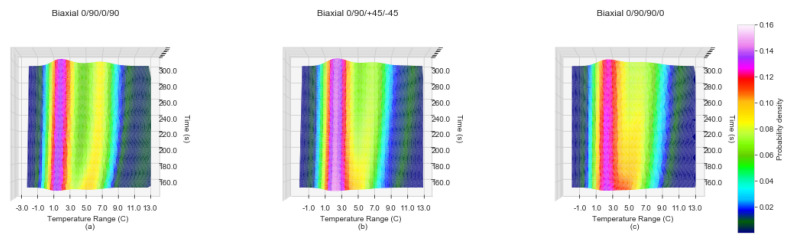
Kernel density estimates top down view of laminate samples for temperature changes ranges −3${}^{\circ }$ to 12${}^{\circ }$ for the time period 150 to 300 s (**a**) Biaxial 0/90/0/90, non symmetrical and balanced, (**b**) Biaxial 0/90/+45/−45, non symmetrical and unbalanced, (**c**) Biaxial 0/90/90/0, symmetrical and balanced.

Figure 3a–c shows the ΔT distribution Biaxial samples with lay-ups of 0/90/0/90, 0/90/+45/−45 and 0/90/90/0 respectively. An overall KDE and its median KDE is computed for every second in the 150–300 time window for every sample (the solid line). The multi coloured region is a side view of the tri-surface plot of the density spread of individual samples as shown in Figure 4.

From Figure 3a the balanced non-symmetrical samples of 0/90/0/90 show a bi-modal distribution of temperature change which can be attributed to the fibre orientation interfaces between plies. For the second group of samples, unbalanced non-symmetrical 0/90/+45/−45, the transitions in fibre orientations are not as steep and consequently the second mode is attenuated. Finally, for the last group of samples, balanced symmetrical 0/90/90/0, the transition between the two slopes is even less steep than that of the unbalanced non-symmetrical 0/90/+45/−45. The significant feature of the KDE characteristic is the presence of two clear peaks evolving by the 300 second timestamp for all of the sample runs. The visualisation of the incremental KDE for each fibre orientation of one sample, accentuates the transitions over time. Figure 4a–c displays KDE plots at between 150 and 300 s for one sample of Biaxial 0/90/0/90, Biaxial 0/90/+45/−45 and 0/90/90/0 respectively. The transition into two distinct peaks for Biaxial 0/90/+45/−45 and Biaxial 0/90/90/0 (Figure 4b,c) becomes more evident. The KDE for Biaxial 0/90/0/90 balanced and non-symmetrical in Figure 4a displays two distinct peaks from 150 s to 300 s, with the transition of the secondary peak advancing along the Temperature Range C axis (*x*-axis).

The transition of the bi-modal peaks forming within the differing fibre orientation sample types becomes clear on inspection of the tri-surface plot of all KDE points as a function of time within a 3D plot viewed from above as shown in Figure 4. Figure 4a displays the Biaxial 0/90/0/90 balanced and non-symmetrical sample with two distinct peaks from the start of 150 second time frame. The peaks settle into perpendicular alignment by approximately the 220 s. Within the Biaxial 0/90/+45/−45 unbalanced sample shown in Figure 4b the secondary peak appears to form the 200 s and a perpendicular alignment of the bi-modal peaks does not become apparent until the 240 s. The fibre orientation sample type of Biaxial 0/90/90/0 displays two peaks which remain within close proximity of each other in regards to the *z*-axis (probability density) which do not appear to form a perpendicular alignment within the 150–300 second time period.

## 4. Data Preparation and Comparative Analysis

In order to provide direct comparative analysis, plots were generated between –2 ${}^{\circ }$C and 13 ${}^{\circ }$C at intervals of 0.25 ${}^{\circ }$C, interpolated from the generated KDE plot points via the numpy function (numpy.interp) for all samples. The interpolated points were then used to create KDE as a function of time plots in the same style as in Figure 4. The variation between the *y* axis (Probability Density) can be compared since these plots are created at a fixed *x*-axis (Temperature Range) and *z*-axis (Time (s)) point. The Mean Square Error (MSE) between two samples can also be evaluated as the interpolation creates a 3 dimensional array. The expectation is that when comparing a randomly selected sample to three samples of differing fibre orientation, the lowest MSE would most likely indicate the samples that share the same fibre orientation. To test the hypothesis, the 30 data sets created from the 10 runs of each of the three fibre orientation samples were split randomly into two groups; the first contained 40% of the total sample data sets used as the testing group; the remaining 60% split further into three groups by fibre orientations. The fibre orientation sets were used to create a mean baseline data set for each fibre orientation by generating a mean KDE as a function of time plot. ($\bar{KDE}/t$). The test group data sets were then compared individually against the $\bar{KDE}/t$ of each fibre orientation and ranked green, amber and red, with green designating the lowest and red the highest MSE; the classification approach utilised in the visual representation of each $\bar{KDE}/t$.

**Figure 5:**
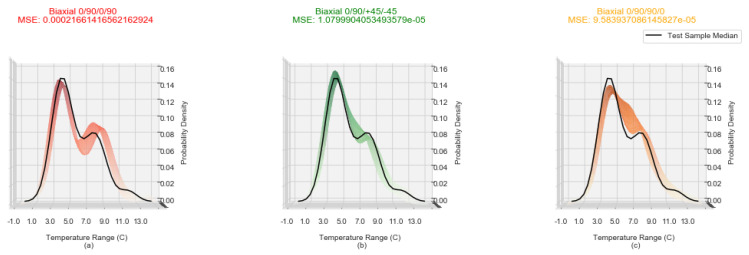
A Biaxial 0/90/+45/−45 sample compared against the $\bar{KDE}/t$ for (**a**) Biaxial 0/90/0/90, (**b**) Biaxial 0/90/+45/−45, (**c**) Biaxial 0/90/90/0.

**Figure 6:**
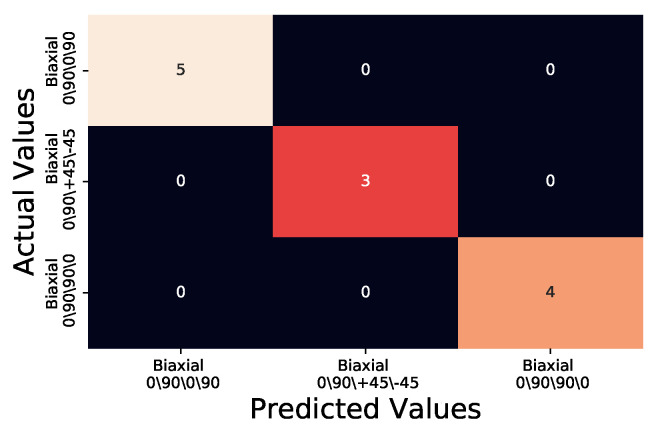
Confusion matrix for testing group samples MSE when compared to $\bar{KDE}/t$.

An example of the representation is shown in Figure 5b where the MSE is the lowest of the three and is thus coloured green, a true positive classification as this is the $\bar{KDE}/t$ generated for Biaxial 0/90/+45/−45. It is also clear on visual inspection that the median of the test sample of unknown fibre orientation (shown in black) sits completely in the $\bar{KDE}/t$ of Figure 5b. In order to quantify the effectiveness of the method the predicted results that the method generated were plotted against the actual values in a confusion matrix (Figure 6). The confusion matrix is traditionally used within machine learning to assess machine learning algorithms ability to correctly predict known test data against training data [21]. As the confusion matrix is designed to present how a Machine Learning algorithm is performing by plotting the predicted values in a grid format against the actual values this method allows for quick visual confirmation as to the performance of the method as well as any increase or decrease between modifications to the method. Within the confusion matrix four classification types can be produced;

- True Positive;
- True Negative;
- False Positive;
- False Negative.

For the Biaxial 0/90/0/90 samples a true positive would be a correct predicted value of Biaxial 0/90/0/90 against the actual value of Biaxial 0/90/0/90. A true negative would be either Biaxial 0/90/+45/−45 predicted value against actual value Biaxial 0/90/+45/−45 or Biaxial 0/90/90/0 predicted value against actual value Biaxial 0/90/90/0. False positives in the case of Biaxial 0/90/0/90 come in the form of predicted values of Biaxial 0/90/+45/−45 or Biaxial 0/90/90/0 against actual values for Biaxial 0/90/0/90. Finally a false negative value for Biaxial 0/90/90/0 is the instance that predicted values for Biaxial 0/90/0/90 are against either Biaxial 0/90/+45/−45 or Biaxial 0/90/90/0 actual values. These classification types are transferable to the other two sample fibre orientation types where Biaxial 0/90/0/90 is swapped with the fibre orientation of which the classification type is being identified.

Figure 6 shows that the small selection of test samples are all correctly classified with their correct fibre orientation, this is seen by the overlap of predicted values and actual values being 100%. Further to the visual display of the confusion matrix, the results can be further analysed to produce precision, recall, F1 scores and overall accuracy of the results. By creating a ratio of predicted values which were correctly identified as the actual values the precision of the method can be quantified (1).

(1) $$Precision=\frac{TruePositive}{TruePositive+FalsePositive}$$

The recall of the results is a quantification of how many of the actual values did the predicted values label as such (2).

(2) $$Recall=\frac{TruePositive}{TruePositive+FalseNegative}$$

An F1 score can also be produced, which is a weighted average of the already calculated Precision and Recall (3). Should the method be tested with an uneven distribution of fibre orientations between test samples the F1 score provides a good indication of comparative performance between test samples.

(3) $$F1=2*\frac{Recall*Precision}{Recall+Precision}$$

Finally the overall accuracy of the method based on the confusion matrix results, which is the ratio of all correct identifications against all values (4) can also be calculated which produces a quantitative value to compare the methods when changes are introduced.

(4) $$Accuracy=\frac{TrueNegative+TruePositive}{TrueNegative+FalsePositive+TruePositive+FalseNegative}$$

As seen in Figure 6 only 12 samples MSE were created against the generated $\bar{KDE}/t$.

### Synthetic Data Inspection

**Figure 7:**
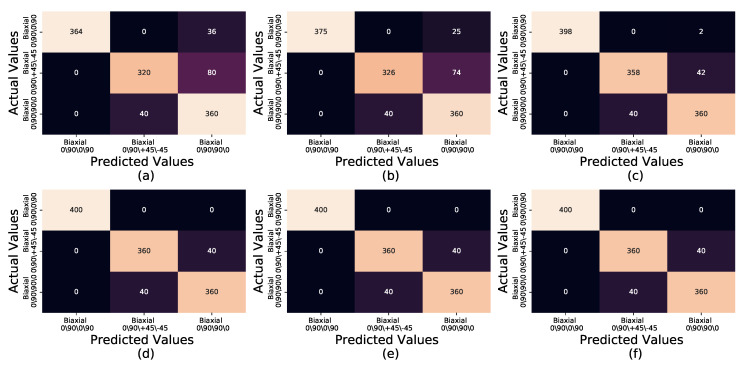
Confusion matrices of Biaxial 0/90/0/90, Biaxial 0/90/+45/−45 and Biaxial 0/90/90/0, for bandwidths: (**a**) 0.6 (**b**) 0.5 (**c**) 0.4 (**d**) 0.3 (**e**) 0.2 (**f**) 0.1.

Due to the COVID19 pandemic of 2020, the authors were unable to obtain further experimentally captured sample readings than the 30 captured prior to restricted access to facilities. In order to produce a larger sample data set an additional six groups of synthetic data sets were generated from the original 30 to further test the accuracy and precision of the method. The data sets were created through the addition of random noise to the experimentally captured thermal profiles. Random Gaussian noise for bandwidths ranging from 0.1 to 0.6 in 0.1 increments were added to each probe reading per second to generate 1200 synthetic samples per bandwidth. To simulate the minor deviation observed in temperature readings between the experimentally captured data sets the random Gaussian noise bandwidth of 0.3 were seen to produce temperatures that fell within the temperature against time profiles of the experimentally captured data sets whilst still providing temperature against time profiles that differed from those experimentally captured. The synthetic data sets were subject to the same MSE comparison as the original experimentally captured test group, against the same $\bar{KDE}/t$ for each fibre orientation. Figure 7a–f displays the confusion matrices for these synthetic data sets; evident is the strong true positive identification of Biaxial 0/90/0/90 at all bandwidths, reaching 100% precision and recall at bandwidth 0.3 continuing through to bandwidth 0.1 (Table 3). The lack of variation in the confusion matrix results between bandwidths 0.1, 0.2 and 0.3 (Figure 7d–f) provide reassurance that the use of a bandwidth of 0.3 does provide a larger simulated data set compared to the experimentally captured data set without producing results that would differ excessively from those expected from experimentally captured data sets. The Biaxial 0/90/+45/−45 has the highest rate of false positives of the three fibre orientations, identifying 80, 74 and 42 samples respectively as Biaxial 0/90/90/0 for bandwidths 0.6, 0.5 and 0.4 respectively. Both Biaxial 0/90/+45/−45 and Biaxial 0/90/90/0 mirror false positives as each other for 40 samples each for bandwidths 0.3 to 0.1, highlighted by identical recall and precision in Table 3 at bandwidths 0.3, 0.2 and 0.1.

A further synthetic data set was created to simulate Biaxial laminates of double the thickness by extending the temperature against time response. The data were generated by inserting a blank time period between each second captured and interpolating the temperature at this time period from the previous and next temperature, in so doing artificially doubling the thermal response time and generating a thermal map representative of laminate plies double the thickness of the experimental samples. Noise from random points of a Gaussian normal process with bandwidth 0.1 was applied to generate 1200 double thickness data points. As before, the synthetic data set was subject to the same MSE comparison as the original experimentally captured test group, against the same $\bar{KDE}/t$ for each fibre orientation.

**Figure 8:**
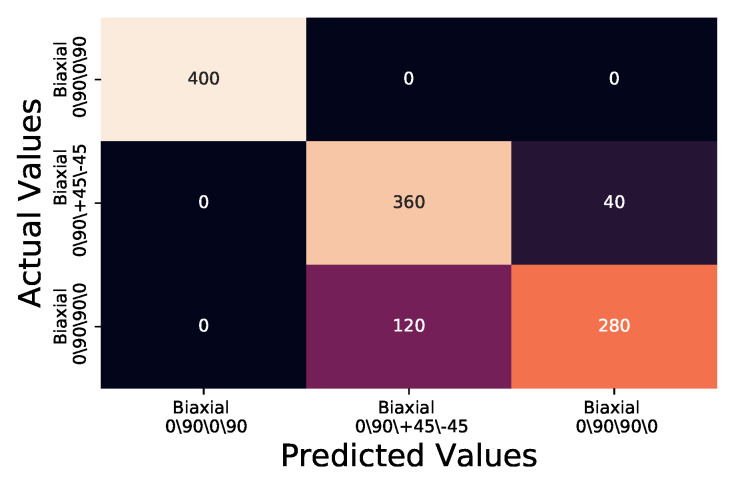
Confusion matrix for synthetically created double thickness samples with 0.1 bandwidth random point of Gaussian normal added for noise MSE when compared to $\bar{KDE}/t$.

The results for Biaxial 0/90/0/90 and Biaxial 0/90/+45/−45 are comparable to those of the regular thickness samples at bandwidth 0.3 as shown in Figure 8 and Table 4. However Biaxial 0/90/+45/−45 has a reduction in precision from 0.9 to 0.5, due to False Positive identification of the Biaxial 0/90/90/0 samples as Biaxial 0/90/+45/−45, also reflected within the precision and recall scores of Biaxial 0/90/90/0 as 0.88 and 0.7 respectively. Overall, the accuracy between the normal thickness and the double thickness samples with random Gaussian normal noise applied at 0.1 bandwidth reduced from 0.93 in the regular to 0.87 in the double thickness samples.

## 5. Conclusions

The feasibility to differentiate and identify fibre orientation within Biaxial laminate CFRP samples using a combination of commercial-off-the-shelf low temperatures and low cost contact sensors has been demonstrated. The approach centres on the application of and then analysis through Kernel Density Estimates with focus on the low temperature change range within the areas of the sample not directly experiencing heat from a heat source. Such an approach allows for the method to be integrated into existing repair/re-manufacture processes through minor modification to existing setup in the form of temperature sensor placement around a heater mat. The resultant dissipation of transient thermal energy within the structure which can then be analysed to yield accurate indications of fibre orientation. The thermal conductivity of the sample is modulated by the fibre orientation.

Utilising synthetic data representative of samples of double the thickness of physical samples, a non symmetrical but balanced sample of Biaxial 0/90/0/90 continued to produce promising indications of high accuracy, recall and precision in true positive identification. This suggests that obtaining experimental data sets from double thickness samples to generate $\bar{KDE}/t$ would improve the identification performance of Biaxial 0/90/90/0 in comparison to the synthetic data. As the approved fibre orientations of aerospace components can be known from the design schematics, and a list of these generated, it is possible to create a $\bar{KDE}/t$ library of fibre orientations that are in use. Should a classification fall outwith the known library, this would provide an indication that the fibre orientation within the structure is not one which is approved and thus should be investigated further.

The method provides a cheap, safe and quick alternative to determine the fibre orientation of composite samples through a routine comparative analysis of KDE plots over a 150 second time period under a 5 min thermal stimulus at low temperature. Results indicate that the method was also successful in classifying synthetically created noisy data representative of regular and double thickness samples with overall accuracy of 0.93 and 0.87 respectively.

## Figures and Tables

**Table 1:** Material thermal properties for biaxial laminate composite plies.

Temperature (K)	Thermal Conductance X Direction (W m${}^{-1}$ K${}^{-1}$)	Thermal Conductance Y Direction (W m${}^{-1}$ K${}^{-1}$)	Thermal Conductance Z Direction (W m${}^{-1}$ K${}^{-1}$)	Specific Heat (J kg${}^{-1}$ K)
218.15	9.3287	9.3287	0.6923	678.26
295.93	11.198	11.198	0.9346	904.35
394.26	12.981	12.981	1.0384	1184.9

**Table 2:** Step heating profile for heat zone set up.

Stage	Time Step (s)	Start Temperature (K)	End Temperature (K)	Ramp Rate (K/s)
Ramp	0–100	298.45	353.15	0.55
Dwell	100–300	353.15	353.15	0.00

**Table 3:** Classification report for synthetic samples, bandwidths 0.6 through to 0.1.

Bandwidth	Sample	Precision	Recall	F1-Score	Accuracy
	Biaxial 0/90/0/90	1.00	0.91	0.95	
0.6	Biaxial 0/90/+45/−45	0.89	0.80	0.84	0.87
	Biaxial 0/90/90/0	0.76	0.90	0.82	
	Biaxial 0/90/0/90	1.00	0.94	0.97	
0.5	Biaxial 0/90/+45/−45	0.89	0.81	0.85	0.88
	Biaxial 0/90/90/0	0.78	0.90	0.84	
	Biaxial 0/90/0/90	1.00	0.99	1.00	
0.4	Biaxial 0/90/+45/−45	0.90	0.90	0.90	0.93
	Biaxial 0/90/90/0	0.89	0.90	0.90	
	Biaxial 0/90/0/90	1.00	1.00	1.00	
0.3	Biaxial 0/90/+45/−45	0.90	0.90	0.90	0.93
	Biaxial 0/90/90/0	0.90	0.90	0.90	
	Biaxial 0/90/0/90	1.00	1.00	1.00	
0.2	Biaxial 0/90/+45/−45	0.90	0.90	0.90	0.93
	Biaxial 0/90/90/0	0.90	0.90	0.90	
	Biaxial 0/90/0/90	1.00	1.00	1.00	
0.1	Biaxial 0/90/+45/−45	0.90	0.90	0.90	0.93
	Biaxial 0/90/90/0	0.90	0.90	0.90	

**Table 4:** Classification report for double thickness synthetic sample, with addition of random Gaussian normal noise bandwidth 0.1.

Bandwidth	Sample	Precision	Recall	F1-Score	Accuracy
	Biaxial 0/90/0/90	1.00	1.00	1.00	
0.1	Biaxial 0/90/+45/−45	0.75	0.90	0.82	0.87
	Biaxial 0/90/90/0	0.88	0.70	0.78	

## Abbreviations

The following abbreviations are used in this manuscript:

CFRP	Carbon Fibre Reinforced Plastic
FRP	Fibre Reinforced Plastic
IR	Infra Red
KDE	Kernel Density Estimate
MRO	Maintenance Repair and Overhaul
MSE	Mean Square Error
OEM	Original Equipment Manufacturer
PID	Proportional Integral Derivative
RTD	Resistence Temperature Detector
